# Chiral Microneedle Arrays With Terahertz Chiroptical Activity With Chiral‐Plasmon‐Chiral‐Phonon Resonance

**DOI:** 10.1002/adma.202521439

**Published:** 2026-06-06

**Authors:** Sang Hyun Lee, Hong Ju Jung, John Kim, Bum Chul Park, Caio V. C. R. da Silva, André F. de Moura, Nicholas A. Kotov

**Affiliations:** ^1^ Department of Electrical Engineering and Computer Science University of Michigan Ann Arbor Michigan USA; ^2^ Center For Complex Particle Systems (COMPASS) University of Michigan Ann Arbor Michigan USA; ^3^ Biointerfaces Institute University of Michigan Ann Arbor Michigan USA; ^4^ Department of Chemical Engineering University of Michigan Ann Arbor Michigan USA; ^5^ Department of Material Science and Engineering Korea University Seoul Republic of Korea; ^6^ Department of Chemistry Federal University of São Carlos São Carlos São Paulo Brazil

**Keywords:** biomolecules, chirality, intrinsic chirality, microneedles, Terahertz chiroptical activity

## Abstract

Microneedle arrays (MNAs) is a rapidly emerging technology with broad biomedical applications in drug delivery and biosensing. With sub‐millimeter dimensions and periodicity, MNAs possess geometries nearly ideal for biomedical devices operating within the terahertz (THz) spectral window. Because chirality is crucial to the function of deposited drugs and surrounding tissues, realizing chiroptical resonances within MNAs could impart new capabilities to microneedle‐based technologies. However, methodologies for fabricating chiral MNAs are largely unknown and their importance remains largerly unrecognized. Here, we present a pathway to arrays of chiral microneedles (ARCHIMs) that exhibit strong and predictable chiroptical resonances in the THz range. These chiroplasmonic microneedles were prepared by glancing angle deposition of two sequential gold layers. ARCHIMs with thin, non‐centrosymmetric caps on each needle exhibit strong chiral plasmonic modes characterized by distinct THz circular dichroism (TCD) bands and polarization rotations as large as 5 degrees. To emulate chiral drugs and biologics, we coated the ARCHIMs with *L*‐ and *D*‐cystine crystals. We found that chiral phonons in the biocrystals resonate with chiral plasmons in the microneedles; their coupling induces handedness‐dependent shifts in the TCD spectra. This photonic effect was quantitatively described using a modified temporal coupled mode theory that incorporates polarization‐dependent resonator parameters. Our findings demonstrate that ARCHIMs provide an effective, tunable, and scalable platform for exploiting chiral light‐matter interactions, opening new opportunities in TCD sensing, chiral diagnostics, chiral phonon detection and THz photonics.

## Graphical abstract

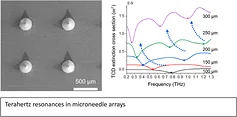

## 1 Introduction

Microneedle arrays (MNAs) have emerged as a transformative biomedical technology for minimally invasive biosensing, enabling real‐time monitoring of metabolites, disease biomarkers, and electrolyte homeostasis in interstitial fluids. Microneedle patches are also being developed for transdermal drug delivery [1, 2, 3], making possible at‐home administration of vaccines, contraceptives, anticancer drugs, and other therapeutics. Recent advances in MNAs also include increasingly sophisticated electronic patches that integrate electrochemical sensing, microfluidic sampling [4, 5, 6, 7, 8], and controll of elution of poorly soluble drugs [9, 10]. In order to (**1**) mitigate surface fouling of microneedle biosensor electrodes and (**2**) monitor biochemical processes at the needle—tissue interface [11, 12, 13], optical techniques have been incorporated to complement traditional electrochemical capabilities [14, 15, 16]. For biosensing using MNAs, the most widely explored optical techniques include fluorescence and Raman scattering spectroscopy [17, 18]. A growing number of studies have integrated MNAs with terahertz (THz) functionalities [19, 20, 21] because the submillimeter dimensions of MNAs are comparable to the wavelengths of THz photons that are also in submillimeter range. Adaptation of MNAs to the THz spectral range offers a promising optical pathway for both drug delivery [19, 20] and patch‐based monitoring [21], expanding the capabilities of microneedles toward multiplexed sensing and diagnostic applications. For example, THz spectroscopy has been used to track transdermal drug delivery via microneedle arrays [20], while THz imaging has enabled tissue monitoring [22]. The enthusiasm for THz‐based sensing within MNA technologies is further fueled by parallel progress in THz metamaterials, which have demonstrated label‐free biosensing with remarkable sensitivity—detecting hemoglobin and early cancer biomarkers via absorptive resonances near 1.1 THz [23, 24].

Given that the vast majority of drugs delivered by microneedles, the biomolecules they detect, and the tissues into which they are inserted are chiral, imparting chiroptical activity to MNAs across any spectral range represents a timely and important research goal. Specifically, THz transmittance absorbance (TA), THz optical rotation dispersion (TORD), and THz circular dichroism (TCD) spectra of microneedles can provide essential information about both drug payload and the local biochemical environment, as the collective excitations of these biomolecular systems predominantly reside in the THz regime. Moreover, many polymers commonly used for drug encapsulation—such as poly‐*L*‐lactic acid (PLLA) —are themselves chiral. Consequently, the ability of TCD spectroscopy to discriminate between the spectroscopic contributions of the drug, analyte, and polymeric carrier could greatly simplify characterization within the rapidly expanding field of theranostic NMAs.

**FIGURE 1:**
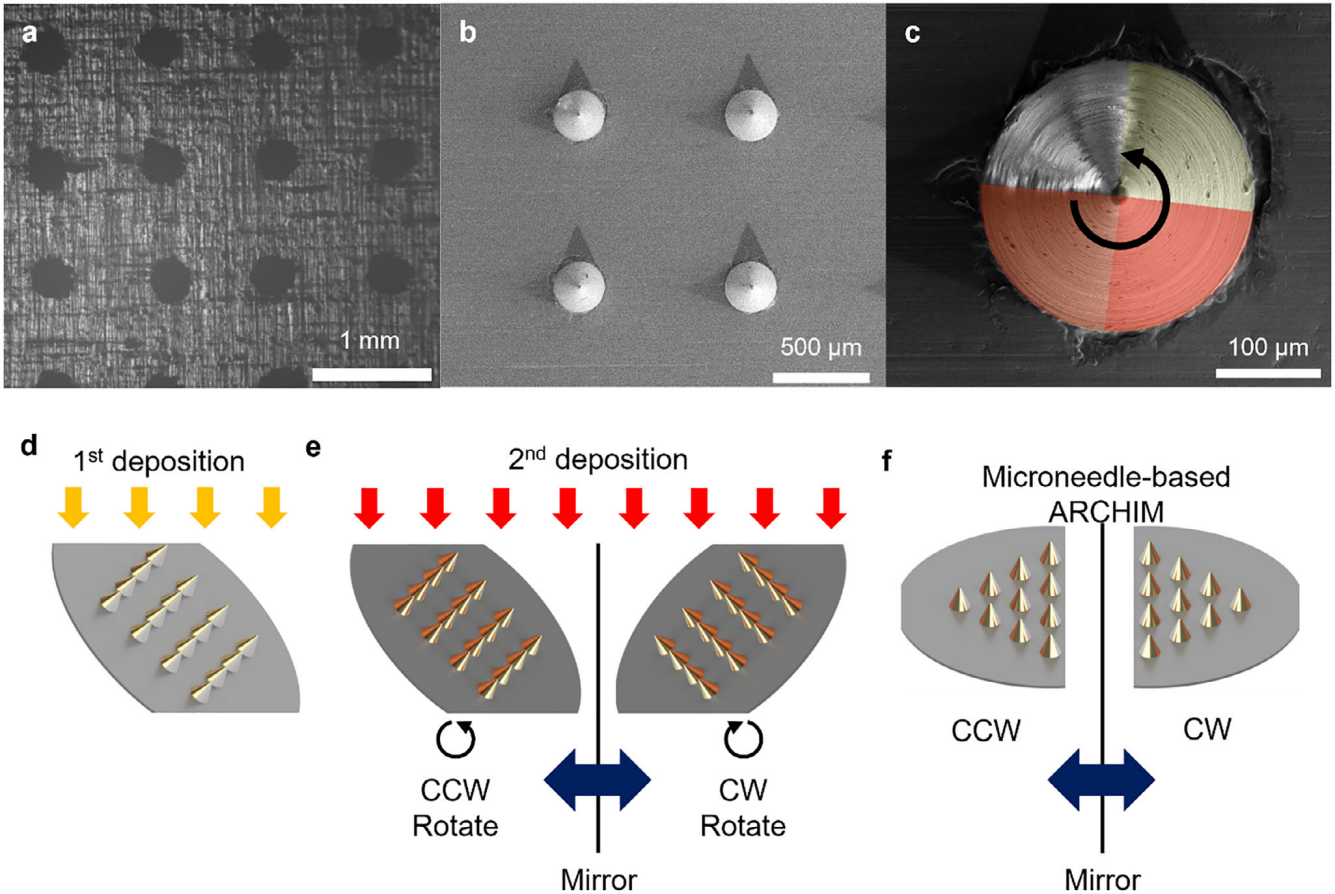
Microscopic images of the microneedle‐based ARCHIM and schematic diagram of the ARCHIM fabrication process. (a) Optical microscopic image of the fabricated CCW ARCHIM. (b) SEM image of multiple microneedles. The visible shadow is due to the glancing angle deposition. (c) SEM image of a single microneedle. The first and second coated metal layers are artificially colored for greater visibility. (d) The first metal layer (Cr 1 nm/Au 3 nm) is deposited on top of the microneedle patch with a glancing angle. (e) The second metal layer (Cr 1 nm/Au 6 nm) is deposited with rotation (CW/CCW) to break mirror symmetry. (f) CW/CCW ARCHIM from a perspective view is presented.

Observations of strong chiroptical responses in the THz range from plasmonic and related nanostructures motivated us to search for chiral resonances in microneedle arrays. Here, we demonstrate that arrays of chiral microneedles (ARCHIMs) can be fabricated by glancing angle metal deposition [25] on commercially available microneedle patches (Figure 1). ARCHIMs exhibit pronounced chiroptical activity under normal incidence of THz light due to chiral plasmons in the gold caps of the needles [26]. Furthermore, the THz resonances of individual microneedles can be tuned to coincide with the chiral phonons of biocrystals [27] deposited onto their surfaces, such as the *L*‐ and *D*‐enantiomers of cystine. We found that coupling between the chiral plasmons and the chiral phonons produces opposite frequency shifts depending on the handedness of the phonon, revealing a previously untapped capability of MNAs for handedness‐resolved THz sensing.

## 2 Results and Discussion

### 2.1 Fabrication of Arrays of Chiral Microneedles

Chiral nano‐ and microstructures represent a rapidly expanding research field at the intersection of chemistry, physics, biology and materials science. Their exceptionally strong circular dichroism and polarization rotation exceed those of conventional organic, inorganic, and biological molecules by several orders of magnitude [28, 29, 30]. Collectively, these effects can be described as giant ellipticity, which has been demonstrated across the UV, visible, red, near‐, medium‐, and far‐infrared spectral regions. Representative examples of chiral nanostructures include nanoparticles (NPs), their hierarchical assemblies, curved nanofilms [25], and a wide range of other architectures composed of gold, silver, and other alloys. These metals, in various compositions, support plasmonic states that can be extended across a single particle, multiple coupled particles, ordered lattices, or engineered surfaces known as metamaterials [31, 32, 33, 34].

We utilized the knowledge about intense chiroplasmonic states to realize TCD‐active MNAs. The fabrication sequence for ARCHIMs incorporating plasmonic nanofilms is illustrated in Figure 1d–f. We started with commercially available Multi‐Dart patches produced by Avarelle. To protect the microneedles from potential damage during fabrication and to ensure chemical stability against solvent‐induced dissolution, a 2 µm‐thick layer of Parylene C was deposited. Glancing angle metal deposition from two directions and in successive steps produced mirror‐asymmetric plasmonic caps [25]. In the first step, a Cr/Au bilayer (1 nm/3 nm) was deposited onto the MNA positioned at a 45 degree glancing angle relative to the incident metal flux, which ensured asymmetric coverage. Before the second deposition, the microneedle patch was rotated by 90 degrees. Counterclockwise (CCW) and clockwise (CW) ARCHIMs were fabricated depending on the direction of patch rotation, resulting in needles with submillimeter scale chiral geometries required for strong resonances with THz waves. The protruding conical geometry of the microneedles enables the formation of out‐of‐plane 3D structures, which significantly enhances their interaction with photons propagating through the patch under normal incidence, with the photon wave vector (*k*‐vector) aligned along the long axes of the microneedles [32].

This simple yet effective method enabled the rapid, controllable, and reproducible fabrication of chiral MNAs. Notably, the thin plasmonic nanofilms allow sufficient transmittance of THz waves, a property essential both for imaging and sensing applications. In contrast, without these nanofilms, additional steps such as lift‐off processing would be required to remove the reflective base [35], which would significantly reduce optical transmittance, an outcome undesirable for biomedical applications.

To confirm the structural integrity of the microneedles and verify successful metal deposition, optical microscopy and scanning electron microscopy (SEM) imaging were performed before and after the deposition process (Figure 1a–c). SEM images confirmed that the conical needle structures remained intact throughout fabrication, with diameters and heights of approximately 250 µm, demonstrating that the encapsulation and deposition steps did not damage the microneedles. The inter‐needle spacing was measured to be 1 mm, which is significantly larger than the THz wavelength used in this study (a few hundred micrometers). Comparison of the pre‐ and post‐deposition SEM images (Figure 1b,c; Figure S1) revealed shadowing effects caused by the incident metal atoms and the partial SEM charging on the non‐conductive microneedle surfaces. Altogether, these image features confirmed the successful realization of chiral features on the submillimeter scale, matching the submillimeter wavelengths of THz photons.

### 2.2 Chiroptical Activity of Microneedles

**FIGURE 2:**
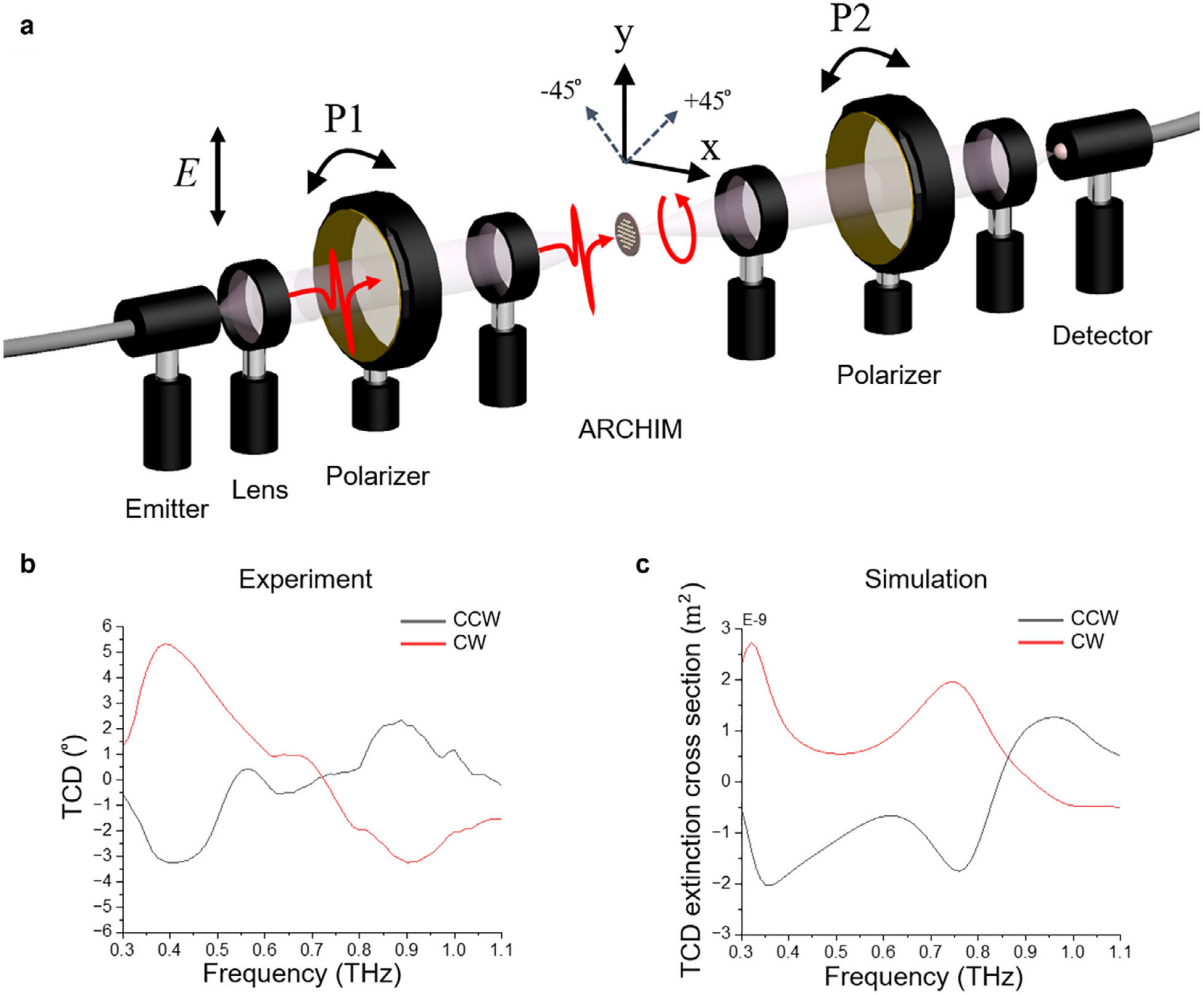
Schematic of THz‐TDP set‐up and the experimental and simulated TCD spectra. (a) Measurements are performed with different polarization‐configurations by rotating two homemade polarizers (P1, P2) to achieve THz polarization activity of the ARCHIM. Each polarizer is aligned ±45 degrees with respect to the linear polarization of the THz pulse from the emitter. Polarizer P1 defines the polarization state of the incident THz pulse, while polarizer P2 selects the detected polarization component. By measuring both parallel and perpendicular configurations between P1 and P2, the full polarization state of the THz field can be reconstructed. (b) Experimental and (c) simulation TCD spectra of the fabricated ARCHIM.

To measure the chiroptical activity of the ARCHIMs in the THz range, we employed a home‐built THz time‐domain spectropolarimeter (THz‐TDP) setup (Figure 2a). Two custom‐made polarizers were integrated into a traditional THz time‐domain spectrometer (THz‐TDS): the first polarizer (P1) was positioned after the THz emitter, and the second (P2) was placed before the detector. This configuration enabled precise measurement of polarization‐dependent responses of the sample. A motorized X‐Y scanning stage was used to control sample positioning, ensuring accurate and statistically reliable THz spectra through pixel‐wise measurements.

Accurate determination of the sample's chiroptical activity in the THz range required a collection of both parallel and cross‐polarized pulses. This was achieved by rotating the two polarizers by ±45 degrees relative to the linear polarization of the THz pulse emitted by the source during measurements [36]. As the sample was scanned on the motorized stage, time‐domain pulses corresponding to different polarization alignment states were recorded for each pixel. For complete extraction of the Jones matrix of the sample, a total of four measurements with distinct polarizer configurations were required. These pulses were subsequently transformed into the frequency domain using fast Fourier transformation (FFT), enabling comprehensive analysis of the sample's polarization response in the THz range. These spectra were then used to calculate key parameters such as TA and TCD, as detailed in the Supplementary Materials.

The non‐zero TCD spectra confirm the emergence of chiroptical activity of the fabricated ARCHIMs, reaching a maximum polarization rotation of ≈ 5 degrees (Figure 2b). This magnitude is nearly 1000 times greater than the typical chiroptical response observed in the visible range using conventional circular dichroism measurements of a single chiral structure [25, 30, 37]. Notably, ARCHIMs with opposite handedness (CCW/CW) exhibit mirror‐symmetric TCD spectra, highlighting that a simple reversal of rotation direction during fabrication produces opposite chiroptical properties. The TCD spectra of ARCHIMs also show a typical bisignate shape, with pronounced chiroptical responses centered at 0.4 and 0.9 THz.

The chiroptical activity of ARCHIMs in THz range was simulated using COMSOL Multiphysics 6.2 software package (COMSOL, Inc.). The microneedle geometry was modeled as a conical structure with a fixed base diameter and height of 250 µm. To replicate the physical characteristics of the fabricated ARCHIMs, varying metal film thicknesses were applied to the conical surface. The scattering extinction cross‐sections for a single microneedle were computed for each circular polarization, and the corresponding TCD spectra were obtained from their differential response. The simulated TCD spectra displayed mirror‐symmetric features that corresponded to the handedness of the ARCHIM and showed a spectral profile consistent with the experimental results (Figure 2c). A slight spectral shift was observed between the simulated and experimental data, which may be attributed to the additional metal deposition on the peripheral regions of the microneedle as well as fabrication tolerances inherent to the glancing angle deposition process.

**FIGURE 3:**
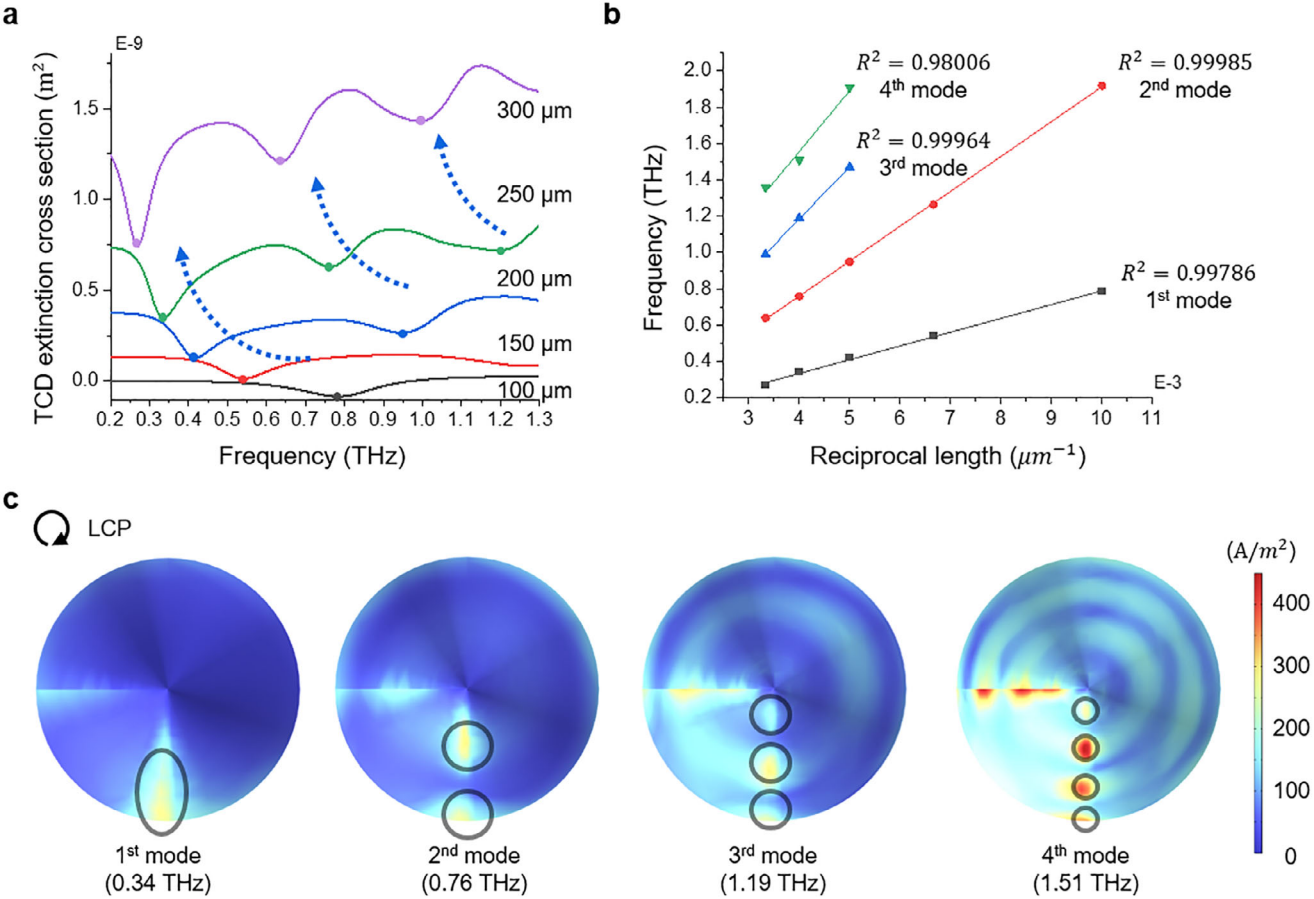
Parametric simulation characterization of the ARCHIM. (a) Simulated TCD spectra depending on the size of the microneedle from 100 – 300 µm. (b) Linear relationship between the peak frequency position and the reciprocal length of the microneedle. (c) Simulated current norm density distribution of each mode with microneedle size of 250 µm.

To investigate the chiroptical behavior of the ARCHIMs, a parametric simulation was performed by systematically varying the microneedle size from 100 to 300 µm, while maintaining equal base diameters and heights. The simulations were performed for the CCW‐handed configuration to examine size‐dependent trends. Because the spacing between adjacent microneedles was insufficient to induce collective lattice resonances in the THz regime (see Supporting Materials for details), both the simulations and their physical interpretations were based on a single microneedle model. The simulated TCD spectra revealed a redshift in the resonance peaks with increasing microneedle size (Figure 3a), consistent with theoretical expectations [38]. Furthermore, larger microneedles exhibited a greater number of resonance peaks within the observed frequency window, indicating the emergence of higher‐order modes and harmonics.

To confirm the relationship between resonance peak positions and microneedle size, the peak frequencies were plotted against 1/*D*, the reciprocal of the needle base diameter (Figure 3b). For all four modes within the analyzed frequency range, linear correlations were observed between reciprocal length and peak frequency, consistent with theoretical predictions [38, 39]. Moreover, higher‐order modes exhibited steeper slopes in these correlations, mirroring the behavior of higher modes in plasmonic nanostructures [40, 41]. These findings confirm that the resonance modes are clearly size‐dependent and can be tuned by changing the microneedle dimensions to achieve desired resonant frequencies. Consequently, the microneedle size serves as a key design parameter for controlling the THz polarization response and optimizing the TCD performance of the ARCHIMs.

To gain deeper insight into the photonic resonance behavior of ARCHIMs, current norm density distributions were analyzed at four resonance frequencies (0.34, 0.76, 1.19, and 1.51 THz) for microneedles with a base diameter and height of 250 µm under left‐handed circularly polarized (LCP) THz wave excitation (Figure 3c). Each resonance exhibited a distinct current distribution, localized along the edges of the metal‐coated regions of the ARCHIM. Notably, the number of high‐current‐density domains increased from one to four as the frequency progressed from the first to the fourth mode. This trend indicates that the resonances correspond to higher‐order standing wave modes supported by the chiral gold caps on the individual needles. These simulation results confirm that the experimentally observed TCD signals originate from the intrinsic plasmonic behavior of single microneedles rather than from the lattice coupling effects, which may occur in a different frequency range.

### 2.3 Coupling of Chiral Plasmons and Chiral Phonons in ARCHIMs

Phonons, the quantized lattice vibrations of crystalline solids [42], can acquire additional properties when manifested in chiral structures [27, 43, 44, 45, 46, 47, 48, 49]. Analogous to chirality in plasmonic systems, chiral phonons represent a rapidly developing field of research, explored through a variety of chiroptical measurements across multiple spectral regions [27, 43, 44, 45, 46, 47, 48]. Notably, chiral phonons [43, 49, 50] have been experimentally measured in a wide range of materials, including nanoparticles [49] and many biological compounds, such as amino acids and amyloid peptides [27, 45]. Note that chiral phonons need to be delineated from axial phonons [50], and both of them need to be delineated from apparent chiroptical activity [44, 49] from linear anisotropic effects in solid state [51].

Both plasmons and phonons can exist within the THz region of the electromagnetic spectrum, creating the possibility for their mutual resonance. In general, energy exchange of plasmons with phonons and excitons is well established in [52, 53, 54]. For example, resonance energy transfer from plasmons to excitons via dipole‐dipole interactions [54, 55] can lead to improvements in photovoltaics [56, 57], photocatalysis [58, 59, 60], and bioimaging [61, 62]. Plasmon‐phonon coupling phenomena have been observed as   Purcell effect [63, 64, 65], Fano resonance [66, 67], surface‐enhanced infrared absorption [68, 69, 70], and polariton excitation [71, 72, 73, 74].

**FIGURE 4:**
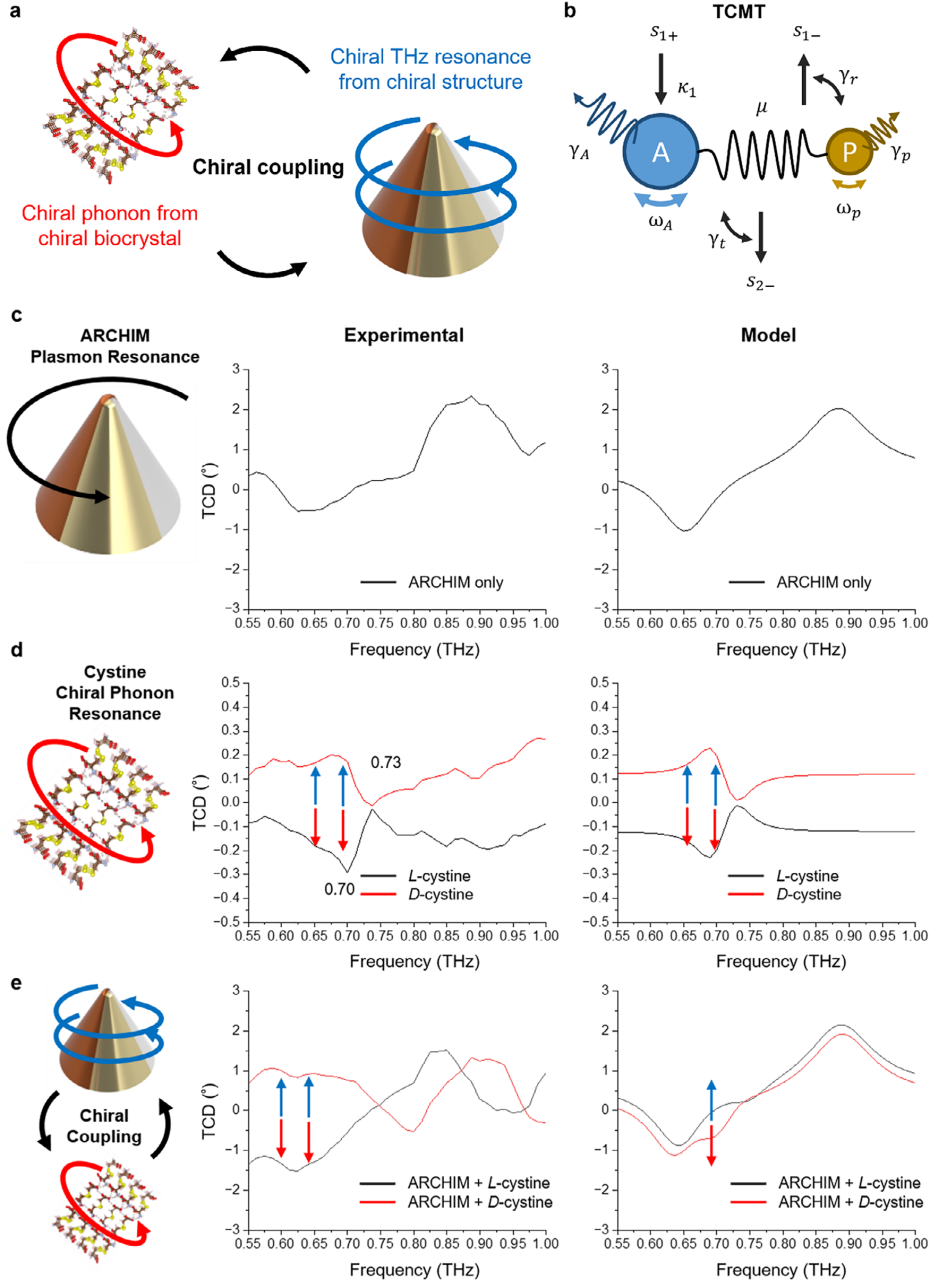
ARCHIM with *L*‐ and *D*‐cystine. (a) Conceptual schematic of chiral coupling between chiral THz plasmonic resonance and chiral phonon from biocrystal. (b) Schematic illustrating TCMT, coupling between ARCHIM chiral plasmon and chiral phonon, describing as harmonic oscillators. Illustration (left), experimental TCD (middle), and theoretical TCD spectra based on TCMT (right) for (c) ARCHIM plasmon resonance, (d) cystine chiral phonon resonance, and (e) coupling between chiral plasmon and chiral phonon.

We hypothesized that chiral plasmons can resonate with chiral phonons. Besides the fundamental significance of this effect, chiral phonons can be very convenient and highly specific means of distinguishing different drugs loaded on MNAs, as well as between isomorphic crystal forms that can strongly impact their pharmacology [75]. The plasmon‐phonon resonances schematically illustrated in Figure 4a [52] can further enhance both the specificity and sensitivity of these systems.

### 2.4 THz Optical Activity of *L‐* and *D‐*Cystine

To evaluate the feasibility of chiralphonon‐chiral‐plasmon resonances in ARCHIMs, we coated the microneedles with *L‐* and *D‐*cystine. These molecules were selected because they display a characteristic chiral phonon peak near 0.72‐0.73 THz (Figure 4c), which can be observed in their TCD spectra [27]. Notably, these frequencies lie in close spectral proximity to the TCD peaks of the ARCHIMs (Figure 2b), which maximizes the plasmon‐phonon coupling. In addition to their optical relevance, both *L‐* and *D‐*cystine, as well as their derivatives possess biomedical importance, serving as an inhibitor of crystal growth in cystinuria [76] and as a treatment for opioid‐induced respiratory depression [77]. These attributes underscore the potential of ARCHIMs as a versatile theranostic platform, capable of simultaneously detecting and differentiating chiral drugs while enhancing light–matter interactions in the THz regime.

Slurries of *L‐* and *D‐*cystine (Sigma‐Aldrich) crystals were prepared by mixing amino acid powders with mineral oil [27]. A phonon peak at 0.71 THz appeared in the TA spectra (Figure S2a). The TCD spectra showed a distinct bisignate response centered at 0.72–0.73 THz, with peaks of opposite polarity for *L‐* and *D‐*cystine (Figure S2b), indicating the presence of chiroptically active vibrational states in cystine enantiomers.

### 2.5 Chiral Phonons in Crystals of *L‐* and *D‐*Cystine

THz vibrational modes of cystine discussed here correspond to infrared‐active modes at the Γ‐point, as probed by transmission‐mode THz‐TDP. In chiral crystals, the equilibrium lattice itself lacks improper rotational symmetry, and Γ‐point vibrational modes can exhibit collective atomic motion constrained by the intrinsic handedness of the crystal. As a result, the corresponding vibrational modes in *L*‐ and *D*‐cystine form mirror‐related enantiomeric pairs, while the time‐reversal operation maps each mode onto itself. This lack of mirror, time‐reversal equivalence gives rise to intrinsic optical activity.

To further substantiate this interpretation, Molecular Dynamics (MD) calculations were carried out to analyze the normal modes and vibrational circular dichroism (VCD) of *L*‐cystine (Figure S4). The calculated eigen‐motions reveal vibrational dynamics constrained by the chiral crystal structure. In addition, the mean rotational strength and mean angular momentum magnitude were evaluated to characterize the helical nature of the atomic trajectories during vibration (Table S3). These results clearly demonstrate the intrinsic chirality of the phonon modes detected by TCD in cystine. Full computational details and mode visualizations are provided in the Supplementary Materials.

To further show that the measured TCD from *L*‐ and *D*‐cystine is not simply a generic consequence of molecular chirality, we also measured another pair of chiral molecules, *L*‐ and *D*‐tryptophan (Figure S3). In contrast to cystine, neither enantiomer of tryptophan exhibits a clear bisignate TCD feature with opposite polarity near the vibrational resonance at 1.45 THz. This comparison indicates that the observed TCD in cystine cannot be explained solely by the natural optical activity of a chiral molecule but is instead associated with phononic response of the crystals. Also note that all the vibrational levels of cystine [78] are all above 500 1/cm (i.e. 15 THz), which is much higher than the observed frequencies in Figure 4d, confirming the attribution of the observed peaks (Figure S3).

### 2.6 Experimental Measurements for THz Resonances in ARCHIM‐Cysteine System

0.5 µL of *L‐* or *D*‐cystine solution (0.01 mg/mL) was drop‐cast directly onto the CCW ARCHIM, and the solvent was allowed to evaporate. SEM images revealed that both enantiomers formed rod‐like cystine crystals on the microneedles (Figure S6). Cross‐polarized optical microscopy further confirms that cystine forms birefringent crystalline domain after drop‐casting, rather than remaining as an amorphous or loosely bound molecular layer (Figure S7). The TCD spectra of the cystine‐coated ARCHIMs split distinctly above and below the baseline spectra of the uncoated ARCHIM, depending on the handedness of cystine (Figure 4e). This splitting can also be detected in the cystine‐coated CW ARCHIM (Figure S8). In addition, TCD spectra of achiral crystal, glycine, drop‐casted ARCHIM were also measured, where it does not produce enantiomer‐dependent TCD shifting (Figure S9). Notably, the TA and TCD spectra exhibited enantiomeric selectivity even at low cystine crystal volumes, an effect not observed when the same amount was drop‐casted onto a bare substrate (Figure S2c, d). These results demonstrate that chiral plasmons from the ARCHIMs and chiral phonons from cystine crystals resonate coherently, confirming the occurrence of chiral plasmon‐phonon coupling. The plasmon‐phonon coupling observed here involves localized angular momentum associated with circular polarization of the plasmonic near field and the lattice vibrational mode at the interface. Also note that our calculations also show that there is a very large number of modes for the cystine crystals. Several of them can be located in the same spectral window in which the ARCHIM plasmon resonance shows its bisignate CD spectrum (Figure 3). Many of them could be in resonance with the plasmonic modes on the microneedles, but chirality of the modes applies specific selection rules favoring only some of them to appear in transmission mode. Other modes can be activated in scattering experimental modality typical of the Raman scattering [79].

The strength and nature of plasmon‐phonon coupling are known to depend on the coupling regime. In general, plasmonic modes supported at simple interfaces as in ACRHIM structures operate in the weak‐coupling regime, where the interaction manifests as spectral reshaping and enhancement. Strong or even ultrastrong coupling regimes can be achieved in alternative architectures that provide enhanced electromagnetic confinement, such as photonic cavities, where plasmon‐phonon hybridization can lead to the formation of polaritonic states. Focusing on ARCHIM, the coupling observed here arises from the interaction between the chiral plasmonic near fields of the MNAs and the collective low‐frequency vibrational modes of the crystal localized near the interface. Within this framework, the effective coupling strength is governed primarily by (a) the spatial overlap between the plasmonic field and the phonon mode, (b) the coverage and morphology of the crystalline layer at the interface, and (c) the strength of the plasmonic mode determined by the ARCHIM geometry. These structure‐dependent factors provide design principles for tuning and controlling chiral plasmon to chiral phonon resonances in the ARCHIM platform.

### 2.7 Temporal Coupled Mode Theory for Chiral Plasmons and Chiral Phonons

To provide a quantitative description of resonance between chiral plasmons and chiral phonons, we employed temporal coupled mode theory (TCMT) [52, 53, 80, 81] in conjunction with the experimental results. As illustrated in Figure 4b, two resonators, which represent the ARCHIM plasmon (A) and phonon (P), are coupled, each characterized by its own resonant frequency. The plasmonic resonator is coupled to the incident (*s*_1 +_), reflected (*s*_1 −_), and transmitted (*s*_2 −_) THz fields, while energy exchange between the plasmonic and phononic resonators is governed by the coupling constant *µ*. This coupled system can be represented by the following equations:

$$\;\frac{\left.da_{A}^{L,R}(t\right)}{dt}=\left(i\omega_{A}^{L,R}-\gamma_{A}^{L,R}\right)\;a_{A}^{L,R}\left(t\right)+i\mu a_{P}^{L,R}\left(t\right)+\kappa_{1}s_{1+}^{L,R}$$

$$\;\frac{\left.da_{P}^{L,R}(t\right)}{dt}=\left(i\omega_{P}^{L,R}-\gamma_{P}^{L,R}\right)\;a_{P}^{L,R}\left(t\right)+i\mu a_{A}^{L,R}\left(t\right)$$

Here, *a* denotes the resonance amplitude, ω is the resonant frequency, γ is the energy loss rate, and κ_1_ is the coupling constant between the incident light and the ARCHIM plasmonic mode in the THz range; the subscripts *A* and *P* refer to the plasmonic and phonon modes of the microneedles, respectively. Unlike conventional TCMT, which typically neglects polarization states and chirality, we modified the model to incorporate circular polarization dependence across all parameters, enabling accurate reproduction of the experimental TCD spectra. The superscripts *L* and *R* denote left‐ and right‐handed circular polarization, respectively.

We first extracted the TCMT parameters using the experimental TCD spectra of CCW ARCHIM without cystine coating (Figure 4c) and of the chiral phonons of *L*‐ and *D*‐ cystine (Figure 4d). Each TCD spectrum was fitted independently, and the TCMT was initially evaluated without inclusion of the coupling constant between the two resonators. Using the parameters from these experimental fittings, (see Supporting Materials), the model‐generated TCD spectra replicated the experimental result. Subsequently, the coupling constant *µ* was introduced into the TCMT framework, after which a similar splitting of the TCD spectra was vividly observed, corresponding to the handedness of the cystine. The residual discrepancy between the measured and modeled coupled response likely arises from additional optical effects beyond the vibrational resonance itself, including dielectric constant of cystine and spatially nonuniform interfacial coupling due to nonconformal cystine coverage on the ARCHIM surface, which are not explicitly captured in our TMCT framework. Nevertheless, this outcome demonstrates that the coupling between chiral plasmons and chiral phonons is responsible for the experimentally observed TCD splitting.

## 3 Conclusions

We have prepared and characterized microneedle‐based chiral plasmonic microstructures with strong chiroptical activity in the THz spectral range. Glancing angle deposition of plasmonic metals convert commercially available achiral microneedle patches into chiral arrays. A pronounced polarization‐selective shift in the TCD spectra was observed, due to coupling of chiral plasmons in the microneedles with chiral phonons in the deposited biomolecules. This phenomenon was quantitatively validated using a modified TCMT that incorporated circular polarization dependence. Our results demonstrate that strong chiroptical activity can be engineered into MNAs, enabling real‐time monitoring of crystalline drug elution and discrimination of chiral isomorphs, both of which are essential for therapeutic efficacy. The ARCHIM platform thus represents a versatile foundation for THz wearable devices, offering spectral tunability for multiple biomedical applications toward both therapeutic and diagnostic capabilities.

## 4 Methods

### 4.1 ARCHIM Fabrication

Parylene C (SCS, Inc.) was deposited onto the microneedle patch by a chemical vapour deposition system (PDS 2035CR, SCS, Inc.) with the thickness of 2 µm for encapsulation. For 2 µm thickness, 5 g of Parylene C dimers was loaded into the vaporizer. No adhesion promoter was used during the deposition process. Any encapsulating material can be used to protect the microneedle patch from further process and dissolution.

The glancing angle metal deposition of chromium (Cr) and gold (Au) was deposited on the Parylene C with an electron beam evaporator (Enerjet evaporator). A home‐made 45 degree substrate holder was used to ensure the glancing angle metal deposition. While normal metal deposition exploit substrate rotation during the deposition, we fixed the sample position without any rotation during the deposition. Cr was deposited before Au for better adhesion of Au during the deposition.

### 4.2 Cystine Solution Preparation

To prepare a fully dissolved solution, 100 mg of *L*‐ or *D*‐cystine (>98%, Sigma) was soaked in 10 mL of deionized water containing 0.3 mL of hydrochloric acid (37%, Sigma) and ultrasonicated for 30 min.

### 4.3 Electromagnetic Simulation

THz responses from ARCHIM, including the TCD extinction cross section, surface current norm distributions, and surface coating with chiral materials, were numerically investigated with commercial software (COMSOL Multiphysics 6.2, COMSOL, Inc.). Among the available physics models, the ‘Electromagnetic Waves, Frequency Domain (emw)’ was employed to calculate the circularly polarized THz beam response from ARCHIM. Because the microneedles were widely spaced, preventing the formation of lattice resonances, the simulations were performed using a single microneedle model.

The 3D structure of the ARCHIM was modeled as a perfect cone with equal height and base diameter. A sphere that covers the whole cone with a radius of 500 µm, along with an inner layer with a thickness of 150 µm, was also added to the geometry of the simulation, which acts as the surrounding environment of the microneedle and the perfect matching layer (PML) for accurate simulation results. The metal layers coated on the needle were realized with the ‘Transition Boundary Condition’ with different thickness with the electrical conductivity being 4.56*10^7 S/m. The extinction cross sections for each circularly polarized THz light were calculated with the polarization of the electric field as (1, *i*) for LCP and (1, −*i*) for RCP to acquire TCD extinction cross section spectra.

## Author Contributions

**S.H.L**. and **N.A.K**. designed the project. **S.H.L**. carried out sample fabrication, THz simulations, and TCMT modeling. **H.J.J**. performed the SEM measurements. **S.H.L**., **J.J.K**., and **B.P**. conducted THz measurement. **C.V.C.R.S**. and **A.F.M**. conducted molecular dynamics simulations. **S.H.L**. and **N.A.K**. wrote the manuscript with contributions from all other co‐authors.

## Conflicts of Interest

The authors declare no conflicts of interest.

## Supporting information




**Supporting File**: adma73622‐sup‐0001‐SuppMat.docx.

## Data Availability

The data that support the findings of this study are available in the supplementary material of this article.
