# Supplementary material for: Chiral Microneedle Arrays With Terahertz Chiroptical Activity With Chiral‐Plasmon‐Chiral‐Phonon Resonance
Source: Adv Mater. 2026 Jun 6;38(48):e21439. doi: 10.1002/adma.202521439 (PMC13508745; doi:10.1002/adma.202521439)
Supplement: Supplementary file 1 [file ADMA-38-e21439-s001.docx]

**Supplementary Information**

**Chiral Microneedle Arrays with Terahertz Chiroptical Activity with Chiral Plasmon-Phonon Resonance**

Sang Hyun Lee^1,3,4^, Hong Ju Jung^2,3,4^, John Kim^2,3,4^, Bum Chul Park^2,3,4,5^, Caio V. C. R. da Silva^6^, André F. de Moura^6^, and Nicholas A. Kotov^2,3,4^*

^1^Department of Electrical Engineering and Computer Science, University of Michigan, Ann Arbor, Michigan 48109, United States.

^2^Department of Chemical Engineering, University of Michigan, Ann Arbor, Michigan 48109-2136, United States.

^3^Center for Complex Particle Systems (COMPASS), University of Michigan, Ann Arbor, Michigan 48109, United States.

^4^Biointerfaces Institute, University of Michigan, Ann Arbor, Michigan 48109, United States.

^5^Department of Material Science and Engineering, Korea University, Seoul 02841, Republic of Korea.

^6^Department of Chemistry, Federal University of São Carlos, 13565-905, São Carlos, SP, Brazil.

*E-mail address: [moura@ufscar.br](mailto:moura@ufscar.br); (A. F. de Moura); [kotov@umich.edu](mailto:kotov@umich.edu) (N. A. Kotov)

**Terahertz Time-Domain Polarimetry (THz-TDP)**

For the generation and detection of the THz pulse, a commercial THz-time domain spectroscopy (THz-TDS, TeraSmart, Menlo Systems) with a 1560 nm wavelength pulsed laser and photoconductive antennas (PCA, Tera15-FC, Menlo Systems) was utilized. The generated THz pulse gets collimated and focused via four convex lenses (TPX50, Menlo Systems) composed of polymethylpentene polymer, which then reaches the target sample and the detector.

On this conventional THz-TDS, two home-made double-stacked wire grid polarizers were added to extract THz chiroptical properties of ARCHIMs. One of the polarizers was placed right after the emitter, and the other was placed right before the detector, and these polarizers were rotatable to control the polarization state of the THz electric field.

To fully determine the THz chiroptical properties of the target sample, we should be able to extract all four elements of the Jones matrix of the sample:

$$T= \left( \begin{matrix} t_{xx} & t_{yx} \\ t_{xy} & t_{yy} \end{matrix} \right)$$

This means that total of four different measurements are needed with different polarizer configurations. By rotating the polarization axis of each polarizer with the angle of ± 45 degrees respect to the *y*-axis, we could achieve four different configurations that enables us to calculate the full Jones matrix of the sample. Fast Fourier transform (FFT) was applied to the measured time-domain THz pulses to obtain results in the frequency domain, which also enabled us to calculate the Jones matrix in the frequency domain.

The complex incident electric field $E_{in}$ and the transmitted electric field through the sample $E_{s}$ can be related as:

$$E_{s}={TE}_{in}$$

Depending on the polarization axis angle of the polarizers, we could express the transmitted electric field as $E_{s,++},E_{s,+-},E_{s,-+},$ and $E_{s,--}$ where $E_{s,(P1,P2)}$ with the ± angle of P1 and P2. The transmittance spectra of the sample and the reference can then be calculated:

$$T_{s}=\frac{\sqrt{{|E_{s,++}|}^{2}+{|E_{s,+-}|}^{2}}+\sqrt{{|E_{s,--}|}^{2}+{|E_{s,-+}|}^{2}}}{2}$$

$$T_{ref}=\frac{\sqrt{{|E_{ref,++}|}^{2}+{|E_{ref,+-}|}^{2}}+\sqrt{{|E_{ref,--}|}^{2}+{|E_{ref,-+}|}^{2}}}{2}$$

where $E_{ref}$ is the transmitted electric field of the reference.

Hence, the THz absorption (TA) can be obtained by:

$$TA=-ln(T_{s}/T_{ref})$$

To calculate THz circular dichroism (TCD), we should be able to calculate the transmitted electric field when the input electric field is circularly polarized. Since the Jones matrix of the sample is known with four different measurements, we can calculate the transmitted electric field with circularly polarized input as follows:

$$E_{sample,RCP}=TE_{in,RCP}=\left( \begin{matrix} t_{xx} & t_{yx} \\ t_{xy} & t_{yy} \end{matrix} \right)\frac{1}{\sqrt{2}}\left( \begin{matrix} 1 \\ i \end{matrix} \right)=\frac{1}{\sqrt{2}}\left( \begin{matrix} t_{xx}+it_{yx} \\ t_{xy}+it_{yy} \end{matrix} \right)$$

$$E_{sample,LCP}=TE_{in,LCP}=\left( \begin{matrix} t_{xx} & t_{yx} \\ t_{xy} & t_{yy} \end{matrix} \right)\frac{1}{\sqrt{2}}\left( \begin{matrix} 1 \\ -i \end{matrix} \right)=\frac{1}{\sqrt{2}}\left( \begin{matrix} t_{xx}-it_{yx} \\ t_{xy}-it_{yy} \end{matrix} \right)$$

Therefore, TCD spectra can be calculated:

$$TCD={tan}^{-1}\left( \frac{E_{R}-E_{L}}{E_{R}+E_{L}} \right)$$

$$E_{R}=\frac{1}{\sqrt{2}}\sqrt{\left| t_{xx}+{it}_{yx} \right|^{2}+\left| t_{xy}+{it}_{yy} \right|^{2}}$$

$$E_{L}=\frac{1}{\sqrt{2}}\sqrt{{|t_{xx}-{it}_{yx}|}^{2}+{|t_{xy}-{it}_{yy}|}^{2}}$$

The capability to extract polarization-resolved responses using the Jones matrix over the broad THz frequency range arises from the time-domain nature of our THz measurements, which is an advantage that is generally unavailable in other optical frequency regimes. In THz-TDS, the electric field is directly measured as a function of time, providing both amplitude and phase information. Consequently, the derived Jones matrix inherently contains complete phase information, enabling the calculation of polarization responses for arbitrary input fields with different phase relationships, without the need for additional experimental measurements.

**Temporal Coupled Mode Theory (TCMT)**

$$\frac{da_{A}^{L,R}(t)}{dt}=\left( i\omega_{A}^{L,R}-\gamma_{A}^{L,R} \right)a_{A}^{L,R}(t)+i\mu a_{P}^{L,R}(t)+\kappa_{1}s_{1+}^{L,R}$$

$$\frac{da_{P}^{L,R}(t)}{dt}=\left( i\omega_{P}^{L,R}-\gamma_{P}^{L,R} \right)a_{P}^{L,R}(t)+i\mu a_{A}^{L,R}(t)$$

Each circular polarization is denoted by superscripts L (LCP) and R (RCP). $a_{A}(t)$ represents the amplitude of the ARCHIM mode, $a_{P}(t)$ represents the amplitude of the phonon mode, $\omega_{A}$ and $\omega_{P}$ represent the resonance frequencies of the ARCHIM and phonon respectively, $\gamma_{A}$ and $\gamma_{P}$ are damping rates for each modes, $\mu$ is the coupling rate corresponding to the energy exchange between two modes, $\kappa_{1}$ is the coupling rate between the incident wave and the ARCHIM mode, and the $s_{1+}$ represents the incident wave. Here, $\gamma_{A}$ is composed of the sum of $\gamma_{r}$, $\gamma_{t}$, and $\gamma_{a}$ which are the damping rates by reflection, transmission, and absorption, respectively. In addition, the coupling rate $\kappa_{1}$ can be also written as $\sqrt{2\gamma_{r}}$.^1,2^

By utilizing a two-port system that considers both the reflection and transmission, the waves of this two-port system can be written as

$$s_{1-}=s_{1+}-\kappa_{1}a_{A}$$

$$s_{2-}=-\kappa_{2}a_{A}$$

where $\kappa_{2}=\sqrt{2\gamma_{t}}$ is the coupling rate between the transmitted wave and the ARCHIM mode.

Therefore, the transmission coefficient can be written as

$$T=\left| \frac{s_{2-}}{s_{1+}} \right|^{2}$$

TCD spectra for the model were then calculated as

$$TCD=A*\left( T^{L}-T^{R} \right)+B$$

where $T^{L,R}$ is the transmission coefficient for LCP and RCP, respectively, A is the amplitude scaling, and B is a baseline offset.

To extract these basic parameters, we fitted each of the equations to the TCD results without coupling. The fitted parameters are shown in **Table 1,** where the chiral phonon was fitted with *D*-cystine.

**Table 1. TCMT fitting parameters for chiral plasmonic resonance in ARCHIM and chiral phonon resonance in *D-*cystine.**

| **Parameter** | **Fitted value** | **Parameter** | **Fitted value** |
| --- | --- | --- | --- |
| $\omega_{A}^{L}$ | 0.653 | $\omega_{P}^{L}$ | 0.725 |
| $\omega_{A}^{R}$ | 0.883 | $\omega_{P}^{R}$ | 0.695 |
| $\gamma_{A}^{L}$ | 0.0353 | $\gamma_{P}^{L}$ | 0.03 |
| $\gamma_{A}^{R}$ | 0.0553 | $\gamma_{P}^{R}$ | 0.03 |
| $A_{A}$ | 1.63 | $A_{P}$ | 0.2 |
| $B_{A}$ | 0.482 | $B_{P}$ | 0.12 |

To calculate *L*-cystine, the values for $\omega_{P}^{L}$ and $\omega_{P}^{R}$ are switched with each other with $B_{P}$ being a negative value.

The coupling constant was introduced to explain the TCD spectra shifting with the cystine-coated ARCHIM. With $\mu$ = 0.03, we could achieve this spectral change in the TCMT. We expect that our proposed chiral TCMT model can be used to explain systems with chiral weak coupling to chiral strong/ultrastrong coupling, such as chiral polaritons.

**Lattice resonance possibility**

For the possibility of collective lattice resonance from the periodic needles, the current center-to-center spacing (1 mm) is too large to support lattice resonance, even in the GHz range. Additionally, the edge-to-edge spacing (0.75 mm) is much greater than the needle diameter (0.25 mm), resulting in insufficient inter-needle coupling. This large discrepancy between spacing and needle size also leads to a low filling factor across the patch, further reducing the likelihood of collective lattice resonance. To achieve lattice resonance, the spacing should be significantly decreased (for THz spectral window, or the needle size should be increased for GHz spectral window.

Prior studies have clearly demonstrated the structural scales to support collective lattice resonances in the THz region.

**Table 2. Structural parameters for eligible THz lattice resonance**

| Reference | Material/Shape | Pitch (μm) | Feature size (μm) | Resonant frequency (THz) |
| --- | --- | --- | --- | --- |
| ^3^ | Au/Square | 140 | 85, 90, 95, 105 | 0.95, 0.91, 0.86, 0.78 |
| ^4^ | Au, Graphene/Square | 140 | 85, 90, 95 | 0.96, 0.89, 0.80 |
| ^5^ | Al/Split ring | 60 | Diameter: 48, Width: 6 | 1.18 (Dipolar mode) |
| ^6^ | Au/Split ring resonator | 90 | Diameter: 60, Width: 6 | 0.83 |

**THz-active structures resonating with THz-active material**

Achiral structures capable of inducing coupling between THz photons and phonons have been reported using several approaches.^7–12^ Due to the subwavelength periodic structures with THz-active materials, most of these approaches operate in the strong coupling regime, where phonons and photons hybridize to form new quantum states called polaritons.^13,14^ The key requirement of these approaches is that the resonant peak frequency from the geometrical structure should be in the range where the phonon peak of the THz-active material lies. With proper matching between these two, the photon and phonon undergoes into the strong coupling regime, resulting phonon polaritons that can be explained by Rabi splitting. In the case of ARCHIM, the chiral structures are not in lattice resonance mode, which cannot induce strong coupling between photon and phonon.

## **L-cystine Supercell Equilibration and Vibrational Circular Dichroism Simulation**

A $5\times5\times1$ supercell of the *L*-cystine crystal was constructed from a .cif file retrieved from the COD database^15^, No. 1513328, taken from the work of MOGGACH and co-workers^16^. Computational procedures were performed using the GROMACS 2021.4 suite^17,18^ and the CHARMM36 force field^19^. Initially, the system underwent energy minimization using the steepest descent algorithm with a maximum force tolerance ($F_{max}$) of 10 kJ·mol⁻¹·nm⁻¹. To equilibrate the simulation box volume and system density, a 1 ns NPT molecular dynamics run was performed at 298.15 K (Thermostat: V-Rescale^20^, τ_T_: 0.1 ps) and 1 bar (Barostat: Berendsen^21^, τ_P_: 1.0 ps, k: 4.6·10⁻⁵ bar⁻¹).

The system's stability was monitored through total energy, volume, and density fluctuations (Supplementary Fig. 5a, b and c, respectively). After reaching equilibrium under the NPT ensemble, the mean volume showed an increase of 3.44% relative to the initial configuration (Supplementary Fig. 5b), with a corresponding mean density decrease of 3.56% (Supplementary Fig. 5c). These variations indicate a proper relaxation of the cell parameters at the target temperature and pressure, ensuring a representative sampling for the subsequent Vibrational Circular Dichroism calculations.

Subsequently, to eliminate residual forces, a second steepest descent minimization ($F_{max}=10$) was conducted, followed by a final refinement using the L-BFGS algorithm with a strict tolerance of 0.1 kJ·mol⁻¹·nm⁻¹. This sequential minimization strategy ensured that the final configuration from the NPT molecular dynamics reached a true potential energy minimum, validated by the absence of imaginary frequencies in the subsequent normal mode analysis (NMA).

Following this, vibrational modes with frequencies up to 1.0 THz were analyzed. For each mode, the averaged rotational strength was determined by computing the dot product between the electric ($\mu$) and magnetic ($m$) dipole moments along the vibrational coordinates, utilizing the fixed partial charges provided by the CHARMM36 force field. This procedure was implemented using a custom Python script leveraging the MDAnalysis library^22^. Each vibrational mode was discretized into 25 frames per cycle to allow for accurate numerical differentiation of atomic velocities. Finally, the Vibrational Circular Dichroism (VCD) spectrum was generated based on the calculated rotational strengths.

## **Simulated Vibrational Circular Dichroism Spectrum and Normal Mode Analysis**

The simulated spectrum (Supplementary Fig. 4a) agrees with the experimental one (**Fig. 4d from the main text**), accurately reproducing both the sign and the spectral position of each band. The modes with the highest rotational strength are marked by red dashed lines, and their corresponding normal modes (Supplementary Fig. 4b,c) reveal coupled vibrations involving multiple *L*-cystine units throughout the supercell, as shown by the displacement vectors (black arrows).

To facilitate visualization, these vibrations are provided as animations in the Supporting Information. For clarity, the normal mode amplitudes were scaled to an effective temperature of 5000 K, and the displacement vectors were magnified by a factor of 30. To minimize visual clutter, only vectors exceeding 30% of the maximum displacement for each mode are displayed. These results demonstrate that the two most intense modes in this region arise from collective vibrations coupled throughout the *L-*cystine supercell, and are inherently chiral.

**Supplementary Fig. 1. SEM images of a bare microneedle patch.**

**Supplementary Fig. 2. THz responses from slurry-state and drop-casted *L*- and *D*-cystine films on quartz.** (a) TA and (b) TCD spectra of slurry state *L-* and *D-*cystine. (c) TA and (d) TCD spectra of drop-casted *L-* and *D-*cystine films on quartz substrate, giving no discrimination between enantiomers.

**Supplementary Fig. 3. THz absorbance (TA) and THz circular dichroism (TCD) spectra of *L*- and *D*-tryptophan**

**Supplementary Fig. 4.** (a) Simulated Vibrational Circular Dichroism (VCD) spectrum in THz range. The two normal modes with the highest rotational strengths are highlighted by red dashed lines at 0.46 and 0.72 THz. Static representations of the modes at (b) 0.46 THz and (c) 0.72 THz are shown, with black arrows indicating the atomic displacement vectors. The yellow, light blue, red, dark blue and white spheres represent the S, C, O, N and H atoms.

**Supplementary Fig. 5. Time evolution of thermodynamic properties during the NPT equilibration phase:** (a) Total energy, (b) system volume, and (c) density.

**Table 3.** **Mean rotational strength (<R>) and the modulus of the mean angular momentum magnitude (<|L|>) for the first modes near the experimental spectral region of interest.**

| Mode | Freq.  (THz) | ⟨R⟩  (e²·Å/ps) | ⟨\|L\|⟩  (u.m.a*A²/ps) |
| --- | --- | --- | --- |
| 4 | 0.356 | 0.000 | 2.589E+03 |
| 5 | 0.405 | 0.000 | 8.800E+03 |
| 6 | 0.423 | -0.001 | 9.857E+04 |
| 7 | 0.461 | -0.026 | 2.228E+04 |
| 8 | 0.722 | 0.036 | 6.086E+04 |
| 9 | 0.780 | 0.004 | 3.327E+04 |
| 10 | 0.814 | -0.001 | 3.988E+04 |
| 11 | 0.824 | 0.003 | 7.343E+03 |
| 12 | 0.827 | -0.001 | 1.462E+04 |
| 13 | 0.840 | 0.005 | 1.325E+04 |
| 14 | 0.844 | -0.001 | 2.192E+04 |
| 15 | 0.852 | -0.001 | 9.978E+03 |
| 16 | 0.852 | -0.001 | 9.488E+03 |
| 17 | 0.866 | -0.001 | 2.272E+04 |
| 18 | 0.875 | 0.006 | 3.247E+04 |
| 19 | 0.880 | 0.001 | 1.689E+04 |
| 20 | 0.882 | 0.002 | 1.627E+04 |

**Supplementary Fig. 6. SEM images of *L*- and *D*-cystine drop-casted on the ARCHIM.** Rod-like cystine crystals are surrounding a single needle.

**Supplementary Fig. 7. Cross-polarized optical microscopy images of *L*- and *D*-cystine.**

**Supplementary Fig. 8. TCD spectra of *L*- and *D*-cystine coated CW-ARCHIM.** Both spectra clearly have shifting of TCD values in the whole frequency range in THz, similar to the cystine coated CCW-ARCHIM.

**Supplementary Fig. 9. TCD spectra of glycine coated CW-ARCHIM.** Only the overall magnitude of TCD spectra is reduced in case of glycine coated ARCHIM, which we attribute to additional damping that modifies the plasmonic mode without introducing a vibrationally resonant coupling.

**Supplementary References**

1.	Agrawal, A. *et al.* Resonant Coupling between Molecular Vibrations and Localized Surface Plasmon Resonance of Faceted Metal Oxide Nanocrystals. *Nano Lett.* **17**, 2611–2620 (2017).

2.	Chang, W. J., Roman, B. J., Green, A. M., Truskett, T. M. & Milliron, D. J. Surface-Enhanced Infrared Absorption Spectroscopy by Resonant Vibrational Coupling with Plasmonic Metal Oxide Nanocrystals. *ACS Nano* **18**, 20636–20647 (2024).

3.	Xu, W. *et al.* Gold Nanoparticle-Based Terahertz Metamaterial Sensors: Mechanisms and Applications. *ACS Photonics* **3**, 2308–2314 (2016).

4.	Xu, W. *et al.* Terahertz biosensing with a graphene-metamaterial heterostructure platform. *Carbon* **141**, 247–252 (2019).

5.	Singh, R., Al-Naib, I. A. I., Koch, M. & Zhang, W. Sharp Fano resonances in THz metamaterials. *Opt. Express* **19**, 6312–6319 (2011).

6.	Geng, Z., Zhang, X., Fan, Z., Lv, X. & Chen, H. A Route to Terahertz Metamaterial Biosensor Integrated with Microfluidics for Liver Cancer Biomarker Testing in Early Stage. *Sci. Rep.* **7**, 16378 (2017).

7.	Xu, R. *et al.* Phonon Polaritonics in Broad Terahertz Frequency Range with Quantum Paraelectric SrTiO3. *Adv. Mater.* **35**, 2302974 (2023).

8.	Jin, X. *et al.* Reshaping the phonon energy landscape of nanocrystals inside a terahertz plasmonic nanocavity. *Nat. Commun.* **9**, 763 (2018).

9.	Mekonen, S. M., Jain, D., Oh, S. & Armitage, N. P. Coupled Metamaterial–Phonon Terahertz Range Polaritons in a Topological Insulator. *ACS Photonics* **11**, 2242–2246 (2024).

10.	Kim, H. S. *et al.* Phonon-Polaritons in Lead Halide Perovskite Film Hybridized with THz Metamaterials. *Nano Lett.* **20**, 6690–6696 (2020).

11.	Jaber, A. *et al.* Hybrid architectures for terahertz molecular polaritonics. *Nat. Commun.* **15**, 4427 (2024).

12.	Kaeek, M., Damari, R., Roth, M., Fleischer, S. & Schwartz, T. Strong Coupling in a Self-Coupled Terahertz Photonic Crystal. *ACS Photonics* **8**, 1881–1888 (2021).

13.	Barra-Burillo, M. *et al.* Microcavity phonon polaritons from the weak to the ultrastrong phonon–photon coupling regime. *Nat. Commun.* **12**, 6206 (2021).

14.	Sidler, D., Schäfer, C., Ruggenthaler, M. & Rubio, A. Polaritonic Chemistry: Collective Strong Coupling Implies Strong Local Modification of Chemical Properties. *J. Phys. Chem. Lett.* **12**, 508–516 (2021).

15.	Mun, J. *et al.* Electromagnetic chirality: from fundamentals to nontraditional chiroptical phenomena. *Light Sci. Appl.* **9**, 139 (2020).

16. Gražulis, S., Chateigner, D., Downs, R. T., et al. Crystallography Open Database (COD): an open-access collection of crystal structures and platform for world-wide collaboration. *J. Appl. Crystallogr.* **42**, 726–729 (2009).
17. Moggach, S., Allan, D., Parsons, S., Sawyer, L. & Warren, J. The effect of pressure on the crystal structure of hexagonal L-cystine. *J. Synchrotron Radiat.* **12**, 598–607 (2005).
18. Berendsen, H. J. C., Van Der Spoel, D. & Van Drunen, R. GROMACS: A Message-Passing Parallel Molecular Dynamics Implementation PROGRAM SUMMARY Title of Program: GROMACS Version 1.0. *Comp. Phys. Comm.* **91**, 43–56 (1995).
19. Abraham, M. J., Murtola, T., Schulz, R., Páll, S., Smith, J. C., Hess, B. & Lindahl, E. GROMACS: High performance molecular simulations through multi-level parallelism from laptops to supercomputers. *SoftwareX* **1–2**, 19–25 (2015).
20. Best, R. B., Zhu, X., Shim, J., Lopes, P. E. M., Mittal, J., Feig, M. & MacKerell, A. D. Optimization of the additive CHARMM all-atom protein force field targeting improved sampling of the backbone ϕ, ψ and side-chain χ1 and χ2 dihedral angles. *J. Chem. Theory Comput.* **8**, 3257–3273 (2012).
21. Buss**i**, G., Donadio, D. & Parrinello, M. Canonical sampling through velocity rescaling. *J. Chem. Phys.* **126**, (2007).
22. Berendsen, H. J. C., Postma, J. P. M., Van Gunsteren, W. F., Dinola, A. & Haak, J. R. Molecular dynamics with coupling to an external bath. *J. Chem. Phys.* **81**, 3684–3690 (1984).
23. Gowers, R. J., et al. MDAnalysis: a Python package for the rapid analysis of molecular dynamics simulations. *Los Alamos National Lab Report* **LA-UR-19-29136**, (2019).
